# Comparative Evaluation of the Impact of Subacute Exposure of Smokeless Tobacco and Tobacco Smoke on Rat Testis

**DOI:** 10.1155/2015/676245

**Published:** 2015-11-08

**Authors:** Jonah Sydney Aprioku, Theresa Chioma Ugwu

**Affiliations:** Department of Pharmacology, Faculty of Basic Medical Sciences, University of Port Harcourt, PMB 5323, Port Harcourt, Nigeria

## Abstract

This study investigated the effects of 30-day exposure to tobacco smoke (TS), smokeless tobacco (ST), and nicotine on reproductive parameters and oxidative biomarkers in prepubertal and adult male rats. Sperm motility was reduced by 77.5 and 89.0% in TS and ST exposed prepubertal rats and 71.1 and 86.4% in adult rats, respectively. Sperm count was also reduced by 64.7 and 89.9% in prepubertal rats and 64.9 and 47.0% in adult rats, respectively. Nicotine decreased sperm motility (82.2%) and count (62.6%) in prepubertal rats but caused no effect in adult rats. There were no changes in sperm morphology; testosterone was decreased, while LH and FSH were increased in exposed rats, when compared with control. Malondialdehyde levels in testes of exposed rats were increased, and GSH, SOD, and catalase were altered. Results indicate that subacute exposure of tobacco products alters sperm characteristics in a rank order of ST > TS > nicotine, which may be linked to increase in oxidative stress in the testis.

## 1. Introduction

Tobacco is known to cause several negative health consequences in both animals and humans [1, 2], and cigarette smoking (tobacco inhalation) and ingestion of smokeless tobacco (e.g., nasal snuff, snus, and moist snuff) are among the major sources of human exposure to tobacco. Aside from the principal biologically active component (nicotine), tobacco products also contain several potentially toxic compounds, including polycyclic aromatic hydrocarbons, cyanide, carbon-monoxide, heavy metals, nitrosamines, and insecticides [3, 4].

Cigarette smoking has been shown not only to cause cancers but also to be associated with increased incidences of chronic obstructive pulmonary diseases, COPDs [5–7], and coronary heart disease [8]. Increasing public awareness of the negative health implications of cigarette smoking, coupled with its restriction in public places by different regulatory bodies, may have controlled the level of smoking in some way. However, this may have at the same time increased the use of smokeless tobacco products as alternatives [9, 10]. In Nigeria, nasal snuff is the most popular form of smokeless tobacco product, and it is consumed in both rural and urban areas not only as alternative to tobacco smoke but also for various other reasons which include medicinal and sociocultural purposes [11]. Furthermore, it has been reported that the use of smokeless tobacco products is becoming more common among young males, and there has been an increase in their production and consumption [12]. This has been partly attributed to the promotion of novel smokeless tobacco products as safer alternatives to smoked tobacco products [10], with the consequence of increasing the potential risk of nicotine poisoning [9]. Unfortunately, there is limited data on the effects of smokeless tobacco, especially on reproductive function because most previous studies have been focused on cigarette smoking [11, 13, 14].

Reproductive dysfunction is a major cause of infertility among couples and tobacco smoke has been shown to cause different forms of reproductive dysfunction in both male and female: low birth weight [14, 15], prenatal and neonatal mortality [16], reduction in uterine blood flow [17, 18], and reduced penile erection [19, 20]. However, the effects of tobacco products on reproductive function continue to be investigated as existing data are not conclusive. Earlier works of Vine et al. [21] and Trummer et al. [22] have reported opposing results on the influence of cigarette smoking on male reproductive hormones. In addition, in spite of the growing knowledge of adverse reproductive effects of smoking on reproduction, it is not certain whether or not nicotine has similar effects and mechanism of action as cigarette smoking on the reproductive system. There are also concerns of the impact of tobacco exposure, particularly smokeless tobacco, on reproductive activity in the young or juvenile male, with the increasing rate of smokeless tobacco consumption in the young.

Earlier studies have shown that cigarette smoke induces apoptosis and degenerative effects on testicular tissues which was associated with increase in oxidative stress [23]. Abdul-Ghani et al. [24] have also shown that cigarette smoke exposure causes impairment of spermatogenesis in rats, which was partly attributed to induction of DNA damage and oxidative stress. In other studies, exposure to cigarette smoke has been reported to induce lipid peroxidation and changes in the oxidative enzyme levels in rat testis [25, 26]. In this study, it is logical to hypothesize that tobacco smoke and smokeless tobacco will produce more deleterious effects than nicotine, attributable to their additional components. In addition, tobacco smoke and smokeless tobacco would alter male reproductive function, mediated through increased oxidative stress, which would be more pronounced in the juvenile animals than the adult [27, 28]. The present study intends to investigate the response of reproductive tissues to subacute exposures of tobacco smoke, smokeless tobacco, and nicotine in prepubertal and adult male rats.

## 2. Materials and Methods

### 2.1. Materials

Benson and Hedges cigarettes (1.0 mg nicotine/stick), locally prepared nasal snuff (16 mg nicotine/g), and nicotine hydrogen tartrate, 98% (BDH Chemicals Ltd., Poole, England), were used.

### 2.2. Animals

Forty-two (42) prepubertal male Wistar albino rats of 5 weeks of age, weighing 80 to 130 g, and 42 adult male Wistar albino rats of 12 weeks of age, weighing 250 to 280 g, were obtained from the Animal House of the University of Port Harcourt. The animals were housed four per cage and fed with standard rat chow and allowed access to tap water* ad libitum*. They were maintained in a well-ventilated room with a 12 h light/dark cycle at room temperature and handled in accordance with the international, national, and institutional guidelines for care and use of laboratory animals as promulgated by the Canadian Council of Animal Care [29].

### 2.3. Experimental Design

The prepubertal and adult rats were each divided into 7 groups containing 6 rats per group and exposed to cigarette smoke, smokeless tobacco, and nicotine. A pilot experiment was done to determine the tolerability of rats to different amounts of cigarette smoke equivalent to 0.25, 0.5, and 1 mg of nicotine using the whole body exposure method as described by Dorman et al. [30] and Wong [31]. Using cigarette containing 1 mg nicotine per stick, the equivalent tobacco smoke doses were 1/4, 1/2, and 1 stick, respectively. None of the doses caused death of animals, so tobacco smoke exposure levels equivalent to 0.5 and 1 mg nicotine were used in the study. Groups I and II of prepubertal or adult rats were exposed to tobacco smoke at target nicotine concentrations of 0.5 or 1 mg daily. To standardize animal exposure, all the exposures were carried out with the same brand of cigarette. Groups III and IV were given smokeless tobacco, nasal snuff (≈0.5 or 1 mg nicotine/kg) daily. Groups V and VI were given nicotine (0.5 or 1 mg/kg) daily. Group VII (control group) animals were allowed to inhale tobacco-free air.

The doses of nicotine used are standard doses in most toxicological investigations [30–32]. Nicotine was serially diluted with normal saline to obtain suitable working concentrations and animals were injected subcutaneously. Nasal snuff (smokeless tobacco) powder was dissolved in distilled water and given by oral gavage. All solutions of nicotine and nasal snuff were stored in foil-wrapped glass bottles at 4°C for no longer than seven days.

### 2.4. Tobacco Smoke Exposure

Whole body exposure is the commonly used method for long-duration exposure studies and for large numbers of test subjects [31]. The model used in this study consists of acrylic plastic cylindrical inhalation chambers (diameter: 30 cm; height: 38 cm; breath: 91 cm). Each chamber has an inner chamber, about 24 cm from the bottom, where the test animals are placed. There is also a wooden lid at the top of the main chambers which ensures minimal leakage of air from the cage and also to restrain the animals.

**Figure 1:**
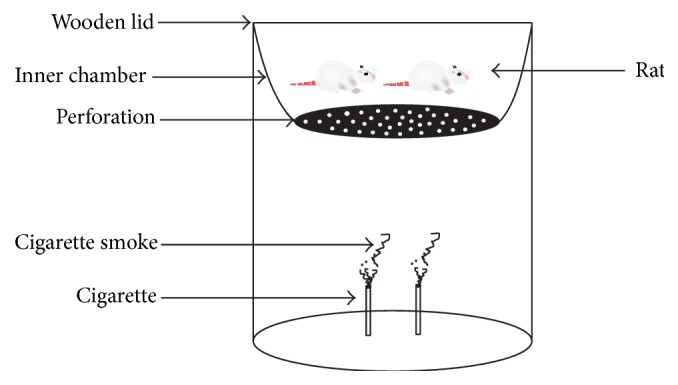
Inhalational chamber showing perforated inner chamber with wooden lid.

The cigarette was lit (1/2 or 1 stick) at the base of the main chamber and the animals (two at a time) were quickly introduced into the inner chamber and the wooden lid was closed and kept for 5–7.5 min or 10–15 min, respectively (each cigarette produces nearly 10–15 min of smoke). The inner chamber has a perforated base, which permits smoke into the inner chamber from the cigarette that is lit at the base of the main chamber (Figure 1). After that, the procedure was repeated with 10-minute interval of rest, and so all the animals received 1/2 or 1 cigarette smoke per day. Each cigarette used contained 1.0 mg nicotine, 10 mg tar, and 10 mg CO. The control group was left free in the interior of chambers and only received compressed air. Tobacco smoke exposures were done under static conditions, and temperatures of the chamber before and during tobacco smoke exposure were monitored (31.5–32°C and 34-35°C, resp.).

At the end of 30 days of treatment, the rats were anesthetized with diethyl ether and sacrificed. Blood samples were collected by cardiac puncture into plain and lithium heparinized tubes. The blood samples were centrifuged at 3000 rpm for 10 minutes and serum was separated and assayed for hormonal levels of testosterone, luteinizing hormone (LH), and follicle stimulating hormone (FSH), using tube-based enzyme linked immunoassay (EIA) technique. Also, the testis was removed along with the epididymis. The caudal epididymis was separated from the testis and lacerated to collect sperm for measurement of sperm indices. Thereafter, the testis was carefully excised, cleared of adhering tissues, and washed in an ice cold 1.15% KCl solution and blotted. Tissues were then homogenized with 0.1 M phosphate buffer (pH 7.2), using a homogenizer. The homogenate was centrifuged at 2500 rmp speed for 15 minutes, and the supernatant was stored at −20°C for estimation of antioxidant enzymes: superoxide dismutase (SOD), reduced glutathione (GSH), and catalase activities and malondialdehyde (MDA) level.

### 2.5. Sperm Analysis

Sperm was placed on a clean dry glass slide and emulsified with equal volume of 1% NaHCO_3_ buffered Tyrodes Lactate solution. Slide was examined under the microscope to measure sperm motility, count, and morphology as described by Baker [33] and Ochei and Kolhatker [34]. Briefly, sperm motility was determined by counting motile and nonmotile spermatozoa in 10 randomly selected fields under the microscope, using 40x objective. Sperm count was done using the improved Neubauer hemocytometer. The Neubauer counting chamber was prepared and charged with diluted seminal fluid and allowed to stand in a moist chamber for 15 minutes. Complete morphologically mature sperm cells were then counted using 40x magnification. Sperm morphology was evaluated by staining sperm smears on microscope slides with a nigrosin-eosin stain after they were air-dried. The slides were examined under the microscope with 100x objective and with oil immersion. The number and percentage of abnormal sperm cells were noted.

### 2.6. Analysis of Oxidative Biomarkers

#### 2.6.1. Superoxide Dismutase (SOD) Enzyme Assay

Superoxide dismutase activity was determined according to the method described by Sun and Zigman [35]. The principle is based on the ability of SOD to inhibit autooxidation of epinephrine determined by the increase in absorbance at 480 nm. To initiate the reaction, testis homogenate (0.02 mL) was allowed to react with 2.95 mL of sodium carbonate buffer (0.05 M, pH 10.2) and 0.03 mL of epinephrine in 0.005 N HCl. The reference cuvette contained 2.95 mL buffer, 0.03 mL of substrate (epinephrine), and 0.02 mL of water. Enzyme activity was calculated by measuring the change in absorbance at 480 nm for 5 min, ∑ = 4020 M^−1^ cm^−1^.

#### 2.6.2. Catalase Enzyme Assay

Catalase activity was assayed colorimetrically at 620 nm and expressed as *μ*moles of H_2_O_2_ consumed/min/mg protein at 25°C, according to the method described by Aebi [36]. The reaction mixture (1.5 mL) contained 1.0 mL of 0.01 M phosphate buffer (pH 7.0), 0.1 mL of testis homogenate, and 0.4 mL of 2 M H_2_O_2_. The reaction was stopped by the addition of 2.0 mL of dichromate-acetic acid reagent (5% potassium dichromate and glacial acetic acid were mixed in 1 : 3 ratio), ∑ = 40 M^−1^ cm^−1^.

#### 2.6.3. Reduced Glutathione (GSH) Enzyme Assay

Reduced glutathione content of the testis as nonprotein sulfhydryls was estimated according to the method described by Sedlak and Lindsay [37]. To the homogenate, 10% tricarboxylic acid (TCA) was added and centrifuged. The supernatant (1.0 mL) was then treated with 0.5 mL of Ellman's reagent (19.8 mg of 5,5-dithiobisnitrobenzoic acid, DTNB, in 100 mL of 0.1% sodium nitrate) and 3.0 mL of phosphate buffer (0.2 M, pH 8.0). The absorbance was read at 412 nm, ∑ = 1.34 × 10^4^ M^−1^ cm^−1^.

#### 2.6.4. Malondialdehyde (MDA) Assay

Malondialdehyde was determined using the method of Buege and Aust [38]. Testis homogenate (1.0 mL) was added to 2 mL mixture of 15% TCA (tricarboxylic acid), 0.37% TBA (thiobarbituric acid), and 0.24 N HCl (hydrochloric acid) reagents (0.37% TBA, 15% TCA, and 0.24 N HCl) in a 1 : 1 : 1 ratio and boiled at 100°C for 15 minutes and allowed to cool. Flocculent materials were removed by centrifuging at 3000 rpm for 10 min. The supernatant was then removed and the absorbance read at 532 nm against a blank. MDA was calculated using the molar extinction coefficient for MDA-TBA complex of 1.56 × 10^5^ M^−1^ cm^−1^.

#### 2.6.5. Statistical Analysis

The results are presented as mean ± SEM for each group (*n* = 6). Differences among groups were analyzed using one-way analysis of variance (ANOVA) followed by Dunnett's multiple range post hoc test for pairwise comparisons. Data were analyzed using GraphPad Prism Version 5.

## 3. Results

### 3.1. Sperm Parameters

**Figure 2:**
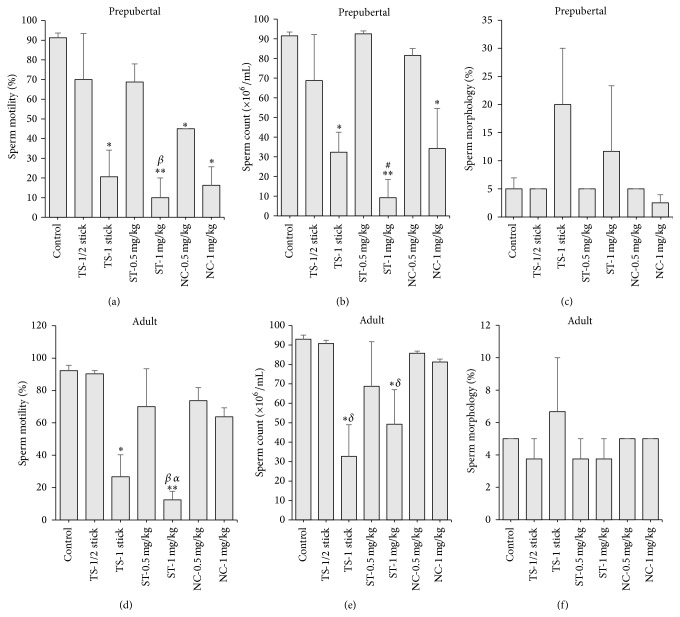
Effects of 30-day daily exposure to cigarette smoke (tobacco smoke, TS), smokeless tobacco (ST), and nicotine on sperm motility, count, and morphology in prepubertal ((a), (b), and (c)) and adult rats ((d), (e), and (f)). Data are expressed as mean ± SEM (*n* = 6). ^*∗*^ *p* < 0.05 compared to control; ^*∗∗*^ *p* < 0.01 compared to control; ^*β*^ *p* < 0.05 compared to TS-1 stick; ^*α*^ *p* < 0.01 compared to NC-1 mg/kg; ^#^ *p* < 0.05 compared to TS-1 stick and NC-1 mg/kg; ^*δ*^ *p* < 0.05 compared to NC-1 mg/kg.

Sperm morphology was not altered in all exposed rats (Figures 2(c) and 2(f)), while sperm motility and counts obtained in tobacco smoke (TS) and smokeless tobacco (ST) exposed prepubertal rats were dose-dependently decreased, compared to non-tobacco exposed control rats (Figures 2(a) and 2(b)). Sperm motility and counts in TS and ST exposed adult rats were also decreased dose-dependently. However, only the results obtained in the rats that were exposed to the higher doses were significantly (*p* < 0.05) different from the controls in both prepubertal and adult rats (Figures 2(a), 2(b), 2(d), and 2(e)). In nicotine treated adult rats, sperm motility, counts, and morphology were not altered, but sperm motility was decreased in all nicotine treated prepubertal rats, while sperm count decreased only in prepubertal rats that received 1 mg/kg, when compared to the control rats (Figures 2(a), 2(b), 2(d), and 2(e)). The levels of reduction of motility produced by the tobacco smoke, smokeless tobacco, and nicotine treatments were 77.5, 89, and 82.2%, respectively, in prepubertal rats and 71.1, 86.4, and 0%, respectively, in adult rats. The corresponding levels of reductions of sperm counts were 64.7, 89.9, and 62.6%, respectively, in prepubertal rats and 64.9, 47, and 0%, respectively, in adult rats. When compared, the motility and sperm counts in prepubertal and adult groups that were exposed to the higher doses of tobacco smoke, smokeless tobacco, and nicotine were also statistically different from each other (Figures 2(a), 2(b), 2(d), and 2(e)).

### 3.2. Oxidative Biomarkers

#### 3.2.1. Lipid Peroxidation (LPO)

**Figure 3:**
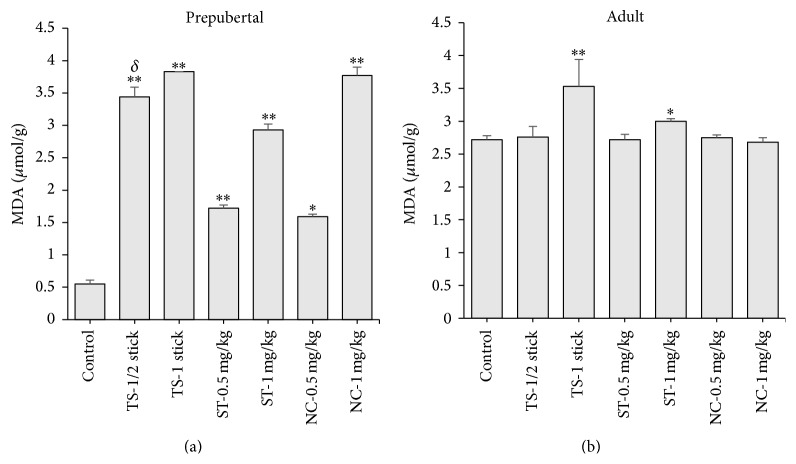
Levels of malondialdehyde (MDA) in testis of prepubertal (a) and adult rats (b) following daily exposure to cigarette smoke (tobacco smoke, TS), smokeless tobacco (ST), and nicotine for 30 days. Data are expressed as mean ± SEM (*n* = 6). ^*∗*^ *p* < 0.05 compared to control; ^*∗∗*^ *p* < 0.01 compared to control; ^*δ*^ *p* < 0.05 compared to ST-0.5 mg/kg and NC-0.5 mg/kg.

In tobacco smoke (TS) exposed prepubertal rat testes, the LPO product, malondialdehyde (MDA) level, was increased (596.4%), compared to non-tobacco exposed control rats (Figure 3(a)). MDA level was also increased in smokeless tobacco (ST) and nicotine exposed prepubertal rats (432.7 and 585.5%, resp.), when compared with non-tobacco exposed control rats (Figure 3(a)). Intragroup comparison showed that the MDA level in TS (1/2 stick) exposed group was different from ST and nicotine (0.5 mg/kg) exposed groups (Figure 3(a)). Furthermore, MDA was elevated in TS and ST exposed adult rats (29.8 and 102.9%, resp.), but there were no significant changes in nicotine treated adult rats, when compared to the controls (Figure 3(b)).

#### 3.2.2. Antioxidants

**Figure 4:**
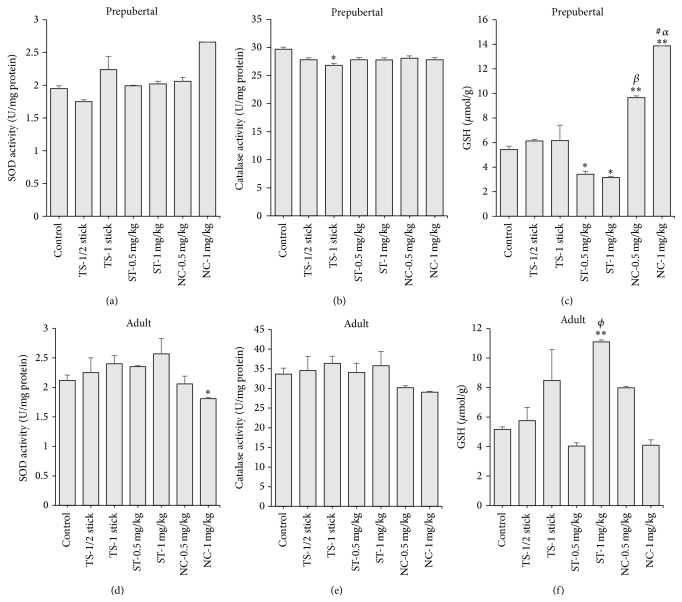
Activities of superoxide dismutase (SOD), catalase, and levels of reduced glutathione (GSH) in testis of prepubertal ((a), (b), and (c)) and adult rats ((c), (d), and (e)) following daily exposure to cigarette smoke (tobacco smoke, TS), smokeless tobacco (ST), and nicotine for 30 days. Data are expressed as mean ± SEM (*n* = 6). ^*∗*^ *p* < 0.05 compared to control; ^*∗∗*^ *p* < 0.01 compared to control; ^*β*^ *p* < 0.05 compared to ST-0.5 mg/kg.; ^#^ *p* < 0.05 compared to TS-1 stick; ^*α*^ *p* < 0.01 compared to ST-0.5 mg/kg; ^*δ*^ *p* < 0.05 compared to ST-0.5 mg/kg and NC-0.5 mg/kg; ^*ϕ*^ *p* < 0.05 compared to NC-1 mg/kg.

In TS exposed prepubertal rat testes, superoxide dismutase (SOD) activity and reduced glutathione (GSH) content were not altered (Figures 4(a) and 4(b)), but catalase was reduced (19.7%), compared to non-tobacco exposed control rats (Figure 4(c)). There were no changes in SOD and catalase activities in ST and nicotine exposed prepubertal rats. GSH was decreased at the higher dose of ST (42.0%), whereas it was increased dose-dependently in nicotine exposed rats, 77.7 and 155.4%, respectively (Figures 4(a), 4(b), and 4(c)). Intragroup comparison showed that the GSH levels in nicotine exposed groups were statistically different from ST and TS exposed groups (Figure 4(c)). Furthermore, SOD was unaffected in TS exposed adult rats (Figure 4(d)), but catalase and GSH were increased, though nonsignificantly (Figures 4(e) and 4(f)). In the ST exposed adult rats, SOD and catalase activities were not altered (Figures 4(d) and 4(e)), but GSH level was elevated, 115.1% (Figure 4(f)). In the nicotine treated adult rats, SOD activity was reduced, 14.6%, but there were no changes in catalase and GSH, when compared to control rats (Figures 4(d), 4(e), and 4(f)). Intragroup comparison showed that the GSH level in ST (1 mg/kg) exposed group was statistically different from nicotine (1 mg/kg) exposed group (Figure 4(f)).

### 3.3. Reproductive Hormones

**Figure 5:**
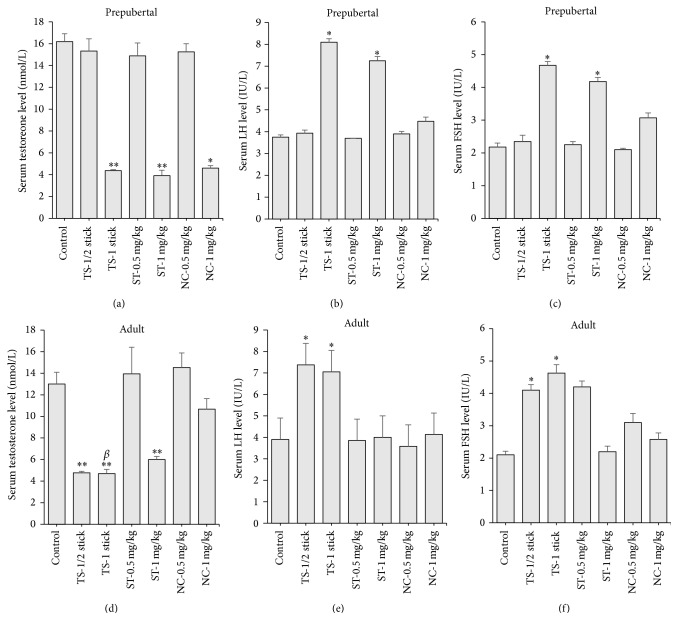
Serum levels of testosterone, luteinizing hormone (LH), and follicle stimulating hormone (FSH) in prepubertal ((a), (b), and (c)) and adult rats ((d), (e), and (f)) following daily exposure to cigarette smoke (tobacco smoke, TS), smokeless tobacco (ST), and nicotine for 30 days. Data are expressed as mean ± SEM (*n* = 6). ^*∗*^ *p* < 0.05 compared to control; ^*∗∗*^ *p* < 0.01 compared to control; ^*β*^ *p* < 0.05 compared to NC-1 mg/kg.

In tobacco smoke, smokeless tobacco, and nicotine exposed prepubertal and adult rats, serum testosterone levels were significantly (*p* < 0.05) decreased but mostly in the groups that were treated with the higher doses (Figures 5(a) and 5(d)). Compared to control rats, the testosterone levels obtained in the exposed prepubertal rats corresponded to 73, 75.9, and 71.6% reductions, respectively, while those obtained in the adult rats corresponded to 63.8, 53.8, and 0% reductions, respectively (Figures 5(a) and 5(d)). In addition, serum levels of LH and FSH were increased in treated prepubertal and adult rats, but this was also mostly observed in the groups that were treated with the higher doses (Figures 5(b), 5(c), 5(e), and 5(f)). The respective serum levels of LH that were obtained in tobacco smoke, smokeless tobacco, and nicotine exposed prepubertal rats were equivalent to 116.0, 93.3, and 19.2% increases (Figure 5(b)), while the serum levels of FSH were equivalent to 114.2, 91.7, and 40.8% increases, respectively (Figure 5(c)). Similarly, the respective serum levels of LH in exposed adult rats were equivalent to 89, 0, and 0% increases, (Figure 5(e)), while those of FSH were equivalent to 120.5, 100.0, and 0% increases, (Figure 5(f)). When compared among the treatment groups, the testosterone level in tobacco smoke (1 stick) exposed adult group was statistically different from nicotine (1 mg/kg) exposed group (Figure 5(d)).

## 4. Discussion

Reproductive organs are highly sensitive to xenobiotics and, in view of the high prevalence of infertility among couples [39, 40], evaluation of xenobiotic exposure to reproductive (male or female) tissues remains pertinent.

Cigarette smoking has been shown to cause several adverse effects on animal and human health, including reproductive toxicity. Previous studies have reported infertility and poor pregnancy outcomes among female smokers [13, 18, 41], as well as alteration of semen parameters in cigarette smoke exposed males [21, 42, 43]. However, similar studies on other tobacco products are limited. Also, the relative impact of tobacco products in juvenile animals has not been well studied.

In this study, tobacco smoke (TS), smokeless tobacco (ST), and nicotine were exposed to prepubertal and adult rats daily at different doses for 30 days and sperm parameters were measured to evaluate the effects of subacute exposure to tobacco products on reproductive function in the male. TS and ST had no significant effect on sperm morphology but reduced sperm motility and also sperm count in both prepubertal and adult rats in a dose-dependent fashion.

Sperm production (spermatogenesis) takes place primarily in the seminiferous tubules in the testis and spermatogenic activity is conventionally assessed by measurement of sperm parameters. Reduction in sperm count and motility observed in exposed rats reflects a reduction in the number and quality of sperm produced in the rats. Further, the observation of these reductions occurring mostly in the rats that were exposed to the higher doses of TS and ST indicates that low levels of TS and ST exposures may not affect testicular activity in the rat. The levels of reduction of the semen indices were observed to be higher in the prepubertal rats in comparison with the adult rats, which also indicates an inverse relationship of toxicity of tobacco products and testicular age. Additionally, ST may produce greater level of testicular toxicity, as the ST induced alterations of sperm motility and count were higher, compared to those of TS. This surprising finding evidences that ST products are not safer alternatives to TS.

Previous reports from several studies have provided contrasting reports on the effect of cigarette smoke on semen parameters. Trummer et al. [22] and de Jong et al. [44] have reported that smoking does not affect semen parameters in humans. On the contrary, Vine et al. [21] and Künzle et al. [43] showed negative effects of cigarette smoking on semen parameters in humans, which is consistent with our results. Unfortunately, the reasons for these different results are not yet fully understood. Some previous studies had attributed the effects of tobacco products mostly to nicotine. Interestingly, our results showed that nicotine treatment had no significant effect on the sperm parameters measured in the adult rats but caused 82.2 and 62.6% reductions in sperm motility and count, respectively, in the prepubertal rats. This provides evidence that the components of tobacco smoke and smokeless tobacco contribute significantly to their testicular effects. Tobacco smoke and smokeless tobacco contain known mutagenic compounds in addition to other compounds that have been shown to be potentially toxic to normal testicular function, including cadmium, lead, and benzo(a)pyrene diol epoxide [4, 45, 46]. The different types and proportions of these components in tobacco smoke and smokeless tobacco products may account for their different degrees of toxicities. Even though there are many publications on the effects of cigarette smoking on semen parameters, limited data exist on the effects of smokeless tobacco, particularly in prepubertal animals. Further, comparative effects of tobacco products and nicotine on semen parameters had been sparsely studied prior to this study, which makes the findings of this study relevant.

Spermatogenesis is regulated by the androgen, testosterone, which is produced and secreted by the Leydig cells of the testis. The secretion of testosterone is in turn regulated by anterior pituitary and hypothalamic factors. Reduction in testosterone levels by tobacco smoke, smokeless tobacco, and nicotine exposures correlates positively with the reduction in sperm count and motility caused by the agents. This was accompanied by elevations of luteinizing hormone (LH) and follicle stimulating hormone (FSH) levels. Earlier, opposing results have also been reported on the hormonal influences of cigarette smoking in males. Ramlau-Hansen et al. [47] reported a positive dose-response relationship between smoking and reproductive hormones (testosterone and LH) in male humans; Trummer et al. [22] reported elevation of serum testosterone in smokers, while Pasqualotto et al. [48] reported absence of any significant difference between cigarette smokers and nonsmokers in levels of FSH, LH, or testosterone. These varying observations may partly be related to differences in the dose and duration of cigarette smoke exposure, animal species, or experimental procedure. The elevations of LH and FSH observed in this study suggest that the negative feedback control of testosterone secretion by the anterior pituitary gonadotropic hormones may be preserved. It thus implies that the observed alteration of sperm characteristics may more likely be due to toxicity to cells in testicular milieu without interference with the hypothalamic-pituitary axis.

From the results, the effects of the tobacco products may be associated with increase in oxidative stress. Oxidative stress in the testis results from an imbalance of the production of free radicals and antioxidant activities. TS, ST, and nicotine caused significant elevations of the lipid peroxidation product, malondialdehyde (MDA), which is consistent with previous results [4, 49]. In addition, reduced glutathione (GSH), catalase, and superoxide dismutase (SOD) were altered. SOD and catalase are essential antioxidant defenses of the body; both catalyze the detoxification of O_2_^−^ reactive radicals and prevent generation of free radicals in the cell. GSH, which contains thiol group, readily interacts with free radicals and is considered to be one of the most important cellular antioxidants to maintain cellular redox state [50, 51]. Testicular tissue is rich in these antioxidants which form effective antioxidant barrier against environmental reactive oxygen species and those generated endogenously by inhaled toxicants [52]. In TS exposed prepubertal rats, SOD activity was not affected, catalase was reduced, and GSH was increased. In both ST and nicotine exposed prepubertal rats, there were no changes in SOD and catalase activities, but GSH was decreased in the former and elevated in the latter. In the exposed adult rats, TS caused no significant alteration of antioxidants; ST increased GSH but did not alter SOD and catalase, while nicotine decreased SOD and did not affect the other antioxidants. Reduced antioxidant activity would increase generation or reactivity of ROS and cause oxidative stress. This compromises testicular function [52] and may contribute to the observed toxic testicular effects of TS, ST, and nicotine. The elevations of the antioxidant levels, observed mostly in exposed prepubertal rats, may reflect the testis adaptation to stress induced by the tobacco products. Similar observations have been reported in the female rat [53].

The results show that subacute exposure to tobacco smoke, smokeless tobacco, and nicotine does not affect sperm morphology but causes dose-related reductions in sperm motility, sperm count, and testosterone, occurring more in prepubertal rats than adult rats. These effects are most pronounced with smokeless tobacco and least pronounced with nicotine, and their mechanism of toxicity may be linked to increase in oxidative stress in the testis. It is, however, important to note that the data in the present study did not address whether or not these effects are reversible, so, there is need for further studies to evaluate the reversibility of these effects.
